# Serum Paraoxonase 1 Activity Status in Patients with Liver Disorders

**DOI:** 10.4103/1319-3767.61232

**Published:** 2010-04

**Authors:** Vivekananda Kedage, Manjunatha S. Muttigi, Mahesh S. Shetty, Renuka Suvarna, Soumya S. Rao, Chitralekha Joshi, Mungli Prakash

**Affiliations:** Department of Biochemistry, Kasturba Medical College, Manipal, India

**Keywords:** Acute viral hepatitis, cirrhosis, falciparum malaria, leptospirosis, sepsis, left ventricular failure, liver disease, paraoxonase 1

## Abstract

**Background/Aim:**

Paraoxonase 1 (PON1) is an esterase, exclusively synthesized by liver. The present study has two objectives: to determine the PON1 activity status in various disorders associated with hepatocellular damage and to correlate the changes of PON1 activity with the standard liver function and fasting lipid profile tests in these disorders.

**Patients and Methods:**

The study groups consisted of 95 patients with liver diseases including acute viral hepatitis (14), cirrhosis with portal hypertension (33), leptospirosis (14), sepsis and multi organ failure (15), left ventricular failure (9), and falciparum malaria (10); and 53 healthy controls. Serum PON1 activity was measured manually using spectrophotometer. Liver function test parameters and fasting lipid profile were performed in clinical chemistry auto analyzer (Hitachi 912).

**Results:**

The serum PON1 activity in patients with acute viral hepatitis and sepsis decreased significantly (*P*<0.001) and moderately in falciparum malaria (*P*<0.05). However, in patients with cirrhosis, leptospirosis and left ventricular patients, its activity did not change significantly. On applying Pearson correlation, serum PON1 activity correlated positively with high-density lipoprotein-cholesterol (HDL-C) in patients with sepsis (r=0.633, *P*<0.05), left ventricular failure patients (r=0.814, *P*<0.05) and negatively with acute viral hepatitis patients (r=– 0.528, *P*<0.05).

**Conclusion:**

PON1 activity has decreased significantly in acute viral hepatitis, sepsis with multi organ failure and falciparum malaria patients. Determination of PON1 activity may serve as a useful additional test in assessing these conditions.

Paraoxonase (aryldialkylphosphatase, EC 3.1.8.1) is a serum esterase. It is a xenobiotic enzyme which hydrolyzes organophosphorous compounds such as paraoxon, unsaturated aliphatic esters, aromatic carboxylic esters etc.[1]*PON1*, *PON2*, and *PON3* are the three members of paraoxonase family, located on chromosome 7q21.3-22.1.[2] Along with apolipoprotein A1 (apoA-I) and clusterin (apolipoprotein J), PON1 is associated with high-density lipoprotein (HDL).[3] This association contributes to the protection against low-density lipoprotein (LDL) oxidation.[1] Assessment of hepatocellular injury and biliary tract disorders include conventional markers such as, alanine and aspartate aminotransferases (AST, ALT), alkaline phosphatase (ALP). Further, hepatic synthetic function can be relatively measured by determining albumin levels in serum.[4] Due to longer plasma half-lives of aminotransferases, changes in hepatocellular damage are not associated with proportional changes in enzyme levels in plasma during acute liver diseases.[4] Hence in these cases more specific tests such as liver biopsy of the affected tissue have to be performed to confirm the suspected cause of the disease.[1]

The liver plays a key role in the synthesis of serum PON1 and the gene expression has been confined only to the liver.[5,6] Also, properties shared by hepatic and serum PON1 are identical, as elucidated by *in vitro* biochemical tests.[7] Hence these observations raise a question on the utility of the measurement of serum PON1 activity as an index of liver function status. Preliminary studies stated serum arylesterase activity decreased significantly in patients with liver cirrhosis.[8–10] Further, Sorenson *et al*. concluded that arylesterase and PON1 activities are functions of a single enzyme.[11] Previous investigators have measured PON1 activity in serum of patients with chronic liver diseases such as alcoholic liver disease, hepatitis, cirrhosis and found that its activity decreased significantly.[1,12–15] Kilic *et al.* have demonstrated that serum arylesterase activity decreased significantly in chronic hepatitis patients.[15] Xu *et al*. have shown previously decreased PON1 activity in chronic liver diseases was significantly increased after successful liver transplantation.[16] PON1 has been shown to protect liver damage by alleviating CCl_4_ induced oxidative stress.[17] Further, Marsillach *et al.* have reported that PON1 protects hepatocytes against inflammation, fibrosis and liver disease.[18]

The current study was undertaken with two objectives. Firstly, to determine the PON1 activity status in various disorders associated with hepatocellular damage. Secondly, to correlate the changes of PON1 activity with the standard liver function and fasting lipid profile tests in these disorders.

## PATIENTS AND METHODS

### Subjects

The study consisted of a total of 148 subjects, 95 patients with liver diseases and 53 healthy controls. Based on the cause for liver disease, patients were divided into six groups [Table 1]. Liver disease was diagnosed based on clinical evidence, radiography, and laboratory investigations. The healthy controls were not on any kind of prescribed medication or dietary restrictions. The demographic and other biochemical data are depicted in Tables 2 and 3. Informed consent was taken from all subjects involved in the study and this study was approved by institutional review board, Manipal University, India.

**Table 1:** Division of patients into six groups based on cause of liver disease

**Groups**	**Number of patients**
Group I–acute viral hepatitis	14
Group II–cirrhosis with portal hypertension	33
Group III–leptospirosis	14
Group IV–sepsis and multi organ failure	15
Group V–left ventricular failure	9
Falciparum malaria	10

**Table 2:** Results of standard liver function tests of healthy controls and patients with liver diseases

	**Healthy controls (n=53)**	**Acute viral hepatitis (n=14)**	**Cirrhosis with portal hypertension (n=33)**	**Leptospirosis (n=14)**	**Sepsis (n=15)**	**Left ventricular failure (n=9)**	**Falciparum malaria (n=10)**
Total bilirubin (mg/dL)	0.80±0.28	8.22±6.01**	4.64±4.19**	3.62±3.2	2.10±0.69	1.57±1.18	3.88±1.8
Direct bilirubin (mg/dL)	0.34±0.16	4.9±4.06**	2.8±2.5**	3.12±2.32	0.88±0.69	0.6±0.5	2.5±1.21
AST (U/L)	18.2±4.01	244±105.7**	104.82±74.92*	219.4±209.1**	227.5±184.3**	52±15.21	85.20±15.8**
ALT (U/L)	15.02±3.9	219.6±88.2**	65.82±54.92*	116.3±84.4**	151.5±106.2**	48±31.01	87.4±46.6*
ALP (U/L)	75.7±2.41	167±62.75*	155.5±62*	279±186.9**	223.4±158**	175±125.3	209±33.53**
Total protein (g/dL)	7.14±0.29	7.45±0.65	7.61±0.9	7.12±1.05	5.5±0.17**	6.30±0.8	5.88±0.72**
Albumin (g/dL)	3.64±0.23	3.7±0.77	3.26±0.86	3.25±1.12	2.24±0.37**	2.76±0.45*	2.66±0.32**
Globulin (g/dL)	3.5±0.17	3.52±0.08	4.38±1.09**	3.87±0.75	3.26±0.44	3.6±0.49	3.22±0.94

** *P*<0.001 compared to normal controls

* *P*<0.05 compared to normal controls; Data expressed as mean±SD

**Table 3:** Demographic, fasting lipid profile and PON1 status of healthy controls and patients with liver diseases

	**Healthy controls (n=53)**	**Acute viral hepatitis (n=14)**	**Cirrhosis with portal hypertension (n=33)**	**Leptospirosis (n=14)**	**Sepsis (n=15)**	**Left ventricular failure (n=9)**	**Falciparum malaria (n=10)**
Age (years)	34±12	47±11	39±9	39±6	35±8	36±11	34±7
Sex (M/F)	31/22	9/5	19/14	6/8	11/4	6/3	6/4
TC (mg/dL)	154.5±16.27	137±72.57	97.65±56.63**	124.41±49.75	143.54±91.66	78.15±30.9*	101.14±13.71
HDLC (mg/dL)	50.14±4.21	68.5±75.7	11.27±13.66**	6.6±5.06**	23.16±13.6*	4.8±1.7**	7.84±6.21**
TAG (mg/dL)	70.02±13.23	130.13±130	97.13±91.6	176.34±87**	136.61±63.67	130.52±86.2	318.37±126.59**
LDLC (mg/dL)	90.8±14.57	124.5±90.24	80.33±61.98	76.44±32.29	135.53±121.7	64.65±24.47	74.35±21.86
RATIO (mg/dL)	3.07±0.38	5.06±4.29	10.59±6.34**	8.44±4.52**	9.46±2.74**	9.8±3.17**	6.32±2.07
PON1 (mg/dL)	141.97±8.27	58.6±22.34**	126.47±104.87	86.8±32.43	53.76±25.81**	134.45±102	76.16±27.83*

** *P*<0.001 compared to normal controls

* *P*<0.05 compared to normal controls; Data expressed as mean±SD

### Samples and reagents

Blood samples (5 mL) were drawn into plain vacutainers from the antecubital veins of healthy controls and patients. The blood was allowed to clot for 30 minutes and centrifuged at 2000g for 15 minutes for clear separation of serum. All the assays were performed immediately after the clear separation of serum. Paraoxon was obtained from Sigma Chemicals Company (St Louis, MO, USA). All other reagents were of analytical grade, obtained from Merck India.

### Biochemical determinations

#### Paraoxonase assay

PON1 was estimated spectrophotometrically by the method described elsewhere with modifications.[19] Briefly, the assay mixture consists of 500 µl of 2.2 mmol/l paraoxon substrate in 0.1 mol/l Tris-HCl buffer, pH 8.0 containing 2 mmol/l CaCl_2_ and 50 µl of fresh serum. After mixing the contents, kinetic measurements were taken immediately at every minute for five minutes, at 405 nm at 25°C. First absorbance reading is taken as 0-minute reading and subsequent absorbance readings were taken as one-minute to four-minute readings. Corrected absorbance readings were obtained by subtracting 1 minute reading with 0 minute reading, likewise, the latter minute reading was subtracted with the previous minute readings. The mean absorbance was calculated. Mean absorbance was used to determine PON1 activity, and standard graph plotted using 1 mM *P*-Nitrophenol. PON 1 activity was expressed in international units (IU). One IU was defined as 1 µmol of p-nitrophenol formed/min/L at 25°C.

#### Standard liver function and fasting lipid profile tests

Serum total and direct bilirubin, aspartate transaminase (AST) and alanine transaminase (ALT), alkaline phosphatase (ALP), total protein (TP), albumin, globulin total cholesterol (TC), high density lipoprotein-cholesterol (HDLC-C), triglycerides (TAG) levels were determined using clinical chemistry analyzer (Hitachi 912). All reagent kits were obtained from Roche Diagnostics, India. LDL-cholesterol (LDL-C) values were derived from Friedewald formula.[20] TC/HDL-C ratio was calculated.

### Statistical analysis

Statistical analysis was performed using the statistical package for Social Sciences (SPSS-16, Chicago, USA). The results were expressed as mean±standard deviation (SD). A *P*-value <0.05 was considered statistically significant. One-way analysis of variance (ANOVA) was used to compare mean values in all groups, followed by multiple comparison *post hoc* tests. Pearson's correlation was applied to correlate between the parameters.

## RESULTS

As shown in Table 2, levels of total and direct bilirubin increased significantly in patients with viral hepatitis (*P*<0.001) and cirrhosis with portal hypertension (*P*<0.001), compared to normal controls. AST activity significantly increased in patients with viral hepatitis (*P*<0.001), cirrhosis with portal hypertension (*P*<0.001), sepsis (*P*<0.001) and falciparum malaria (*P*<0.001). Activity of ALT significantly increased in viral hepatitis (*P*<0.001) and sepsis patients (*P*<0.001). ALP activity significantly increased in patients with viral hepatitis (*P*<0.001), cirrhosis with portal hypertension (*P*<0.001), leptospirosis (*P*<0.001), sepsis (*P*<0.001) and falciparum malaria (*P*<0.001). Levels of TP decreased significantly in sepsis (*P*<0.001), falciparum malaria (*P*<0.001), and moderately in left ventricular failure patients (*P*<0.05). Albumin levels decreased significantly in falciparum malaria (*P*<0.001), and moderately in sepsis (*P*<0.05) and left ventricular failure patients (*P*<0.05). Levels of globulins significantly increased in cirrhosis with portal hypertension (*P*<0.001).

As shown in Table 3, TC levels significantly decreased in patients with cirrhosis with portal hypertension (*P*<0.001) and moderately in left ventricular failure patients (*P*<0.05). Levels of HDL-C decreased significantly in patients with cirrhosis with portal hypertension (*P*<0.001), leptospirosis (*P*<0.001), left ventricular failure (*P*<0.001) and falciparum malaria (*P*<0.001) and moderately in sepsis (*P*<0.05). TAG levels increased significantly in leptospirosis (*P*<0.001) and falciparum malaria (*P*<0.001) patients. The ratio TC/HDL-C increased significantly in patients with cirrhosis with portal hypertension (*P*<0.001), leptospirosis (*P*<0.001), sepsis (*P*<0.001) and left ventricular failure (*P*<0.001). PON1 activity significantly decreased in viral hepatitis (*P*<0.001) and sepsis (*P*<0.001) and moderately in falciparum malaria patients (*P*<0.001). On applying Pearson correlation, serum PON1 activity correlated positively with HDL-C in patients with sepsis (r=0.633, *P*<0.05), left ventricular failure patients (r=0.814, *P*<0.05) and negatively with acute viral hepatitis patients (r=– 0.528, *P*<0.05).

## DISCUSSION

When it comes to cardiovascular diseases, serum PON1 has been studied extensively.[21–25] To date, the gene expression of PON1 have been confined only to liver.[5,6] Thus measurement of serum PON1 defines hepatic synthetic function. As shown in Table 2 and discussed in the results section, a wide spectrum of changes in standard liver function indicates hepatocellular damage. In the present study, serum PON1 activity significantly decreased in patients with acute viral hepatitis, sepsis with multi organ failure and falciparum malaria. Previous authors have demonstrated a decrease in PON1 activity in chronic viral hepatitis.[1,15] In this study, we have measured PON1 activity in patients with various liver disorders including acute viral hepatitis. In line with the previous authors, there was decrease in PON1 activity in acute viral hepatitis patients.

Previous authors have proposed two mechanisms to explain the decrease in activity in PON1 in liver disorder patients. First, as there is hepatic dysfunction, it is obvious that there is defective gene expression, which contributes to decreased PON1 in these patients.[1] It has been reported that there was significant decrease in PON1 activity in CCl_4_ induced liver cirrhosis secondary to increased free radical generation.[26] Second, as a consequence of an altered synthesis and/ or secretion of HDL-C, which may be due to impaired lecithin:cholesterol acyl transferase (LCAT) activity.[1] We have observed positive correlation between PON1 activity and HDL-C levels in patients with falciparum malaria and sepsis. In contrast, in patients with acute viral hepatitis, decrease in PON1 activity is not associated with a proportional decrease in HDL-C levels, as there is negative correlation between PON1 activity and HDL-C levels. Several workers have proposed earlier that viral hepatitis is associated with oxidative stress.[27–31] Further, PON1 activity associated with HDL-C in plasma is thought to protect LDL-C oxidation.[15]

A previous study has stated that decrease in PON1 activity in patients with chronic liver diseases such as chronic hepatitis and cirrhosis, was related to degree of liver damage.[1] Recently, Keskin *et al.* also have reported reduced baseline and stimulated PON1 and arylesterase (ARE) activities in patients with chronic liver disease.[32] Contradictory to the above study, we have observed that there was lowered PON1 activity in acute viral hepatitis but normal PON1 activity in patients with cirrhosis. Furthermore, Ferré *et al.* demonstrated that there was a significant change in PON1 activity in patients with cirrhosis but our results are contradictory, as we found no significant change in its activity.[1] In our study, there was no significant decrease in the PON1 activity in patients with cirrhosis, leptospirosis and left ventricular failure patients, but there was a significant decrease in HDL-C levels in these patients. To explain this observation we speculate that the extent of hepatocyte damage in these conditions may not be so severe to significantly decrease PON1 activity. Although the PON1 gene expression reported by previous studies has been confined only to liver,[5,6] significant presence of PON1 activity in these patients, despite largely decreased HDL-C, may raise the possibility of a second source of PON1 in the body, other than liver. However, further research is necessary to substantiate this possibility, prove or disprove it.

In conclusion, PON1 activity has decreased significantly in acute viral hepatitis, sepsis with multi organ failure and falciparum malaria patients. There was no significant change in PON1 activity in cirrhosis, leptospirosis and left ventricular failure patients, but there was a significant decrease in HDL-C levels in these patients.
